# Research on Underwater Target Detection Method Based on APO-DBSCAN Clustering

**DOI:** 10.3390/s26061885

**Published:** 2026-03-17

**Authors:** Shengwen Duan, Gang Bian, Qiang Liu, Pan Xiong

**Affiliations:** Department of Military Oceanography and Hydrographic and Cartography, Dalian Naval Academy, Dalian 116018, China

**Keywords:** APO algorithm, DBSCAN clustering, discrete solution quality control, Eulerian inverse integral, magnetic target localization

## Abstract

To address critical issues in traditional quality control methods for discrete Euler solutions in underwater magnetic target detection—such as excessive filtering of valid solutions during divergence suppression, parameter settings reliant on subjective experience, and insufficient noise resistance—this study proposes a novel approach combining the Artificial Protozoa Optimizer (APO) with DBSCAN clustering. Based on the distribution characteristics of Euler solutions, an optimization objective function incorporating Euler solution residual penalty terms and contour line coefficients was constructed. The APO algorithm identifies DBSCAN clustering parameters that minimize this objective function, thereby enhancing clustering precision and accuracy. This method selects optimal Euler solution sets, enabling high-precision localization of magnetic targets. Simulation and field test results demonstrate that compared to statistical screening methods, the optimized algorithm achieves a 52.52% and 76.33% increase in the retention rate of valid solutions for noise-free and noisy data, respectively, while reducing the retention rate of invalid solutions by 28.57% and 94.21%. In field data, the average deviation from the true center of gravity is reduced by 28.06%.

## 1. Introduction

Underwater target detection holds critical strategic significance in fields such as national defense security, marine resource development, and marine engineering construction. Whether for monitoring underwater vehicles, exploring seabed mineral resources, salvaging sunken artifacts, or inspecting subsea pipelines, accurately locating the spatial position of underwater targets is a core prerequisite for ensuring mission success. With the increasing frequency of marine development activities and the growing complexity of underwater environments, higher demands are placed on the accuracy and efficiency of underwater target detection. As a vital method for underwater target detection, the performance optimization and methodological innovation of magnetic field data inversion and interpretation techniques have garnered significant attention.

The Euler deconvolution method is a classic technique for detecting and locating underwater targets [1,2]. This approach enables semi-automated or fully automated source attribution interpretation under conditions of limited prior information, effectively determining the spatial location and distribution range of sources. It demonstrates significant advantages in scenarios requiring efficient processing of large-scale, multi-objective datasets [3,4]. Currently, This method has become a core and commonly used technique for the inversion and interpretation of gravity and magnetic fields, and is widely applied in multiple fields such as underwater and subsurface magnetic target detection [5], mineral resource exploration [6], and geological and geophysical surveys [7]. At present, when using the Euler deconvolution method to solve for the spatial location of field sources, the sliding Euler window mode is commonly employed, meaning that a single window can only invert the spatial location parameters of a single field source [8]. This processing mode yields a set of Euler solutions for source parameters, creating significant challenges for interpreters in identifying and characterizing source properties. To accurately obtain the unique spatial location parameters for each source, further refinement of these Euler solutions is required—a process defined as “quality control” [9].

To address the issues associated with the quality control of Euler deconvolution, various strategies have been proposed in the literature. Yao et al. (2004) introduced several screening criteria, including horizontal gradient filtering, distance constraint evaluation, and aggregation degree constraint evaluation, which improved the applicability of 3D Euler deconvolution [10]. Wang et al. (2013) adopted the singular value decomposition–total least squares (SVD-TLS) method to reduce the non-uniqueness and instability of Euler solutions caused by improper singular value analysis, and verified its effectiveness through numerical examples [11].

A different line of research focuses on clustering-based filtering. Ugalde and Morris (2010) proposed a density-based clustering method as a screening tool, based on the observation that Euler solutions tend to cluster densely only near field sources while being sparse elsewhere [12]. They used kernel density estimation (KDE) to remove irrelevant solutions and applied fuzzy C-means (FCM) clustering to estimate the geological distribution and strike of anomalous sources. However, this approach fails to effectively distinguish spurious solutions arising from shallow sources or adjacent anomalous bodies [13]. Cao et al. (2012) later introduced an adaptive fuzzy clustering algorithm to cluster Euler solutions obtained from tensor Euler deconvolution for identifying multiple anomalous sources [14]. Nevertheless, its performance deteriorates significantly for non-spherical clusters (e.g., strip-shaped or ring-shaped clusters), and it cannot automatically identify noise points—sparse invalid solutions are forcibly assigned to a cluster and must be manually filtered afterwards.

Reid et al. (2014) proposed filtering spurious solutions based on the clustering characteristics of the solutions, their goodness of fit, and their depth and distance relative to the sliding window [15]. While these methods have advanced the quality control of Euler solutions, they still rely heavily on manually set parameters or suffer from limitations in handling complex data distributions.

In response to these limitations, Bian et al. [16] proposed an effective screening control scheme. By constructing a filtering model combined with distance judgment, cluster merging, and spurious solution elimination, this scheme achieves the refinement of Euler solutions and improves positioning accuracy to a certain extent. However, this method requires manual presetting of multiple parameters such as distance thresholds and confidence levels, which is highly subjective and prone to problems such as over-screening or residual spurious solutions. Li et al. [17] first introduced the DBSCAN density clustering algorithm into the quality control of Euler solutions. Taking advantage of its ability to identify clusters of arbitrary shapes and automatically distinguish high-density valid solutions from low-density spurious solutions, this method realizes the positioning of multiple magnetic targets through outlier removal, cluster analysis, and cluster screening. Nevertheless, this method still relies on manual empirical setting of key DBSCAN parameters, namely the neighborhood radius (Eps) and minimum number of points (MinPts). In complex magnetic anomaly scenarios, insufficient parameter adaptability tends to reduce clustering accuracy, making it difficult to balance the retention rate of true solutions and the elimination efficiency of spurious solutions.

For decades, the core challenges in Euler solution quality control have persisted: (1) excessive reliance on subjective empirical parameters, leading to poor generalization across different scenarios; and (2) inadequate noise robustness and adaptability to complex magnetic anomaly distributions.

Against this backdrop, this study proposes a novel quality control framework for Euler solutions based on APO-DBSCAN clustering, addressing the aforementioned gaps through three key innovations. First, building on the inherent spatial concentration of valid Euler solutions and DBSCAN’s advantage in arbitrary-shaped cluster recognition, we abandon manual parameterization by introducing the Artificial Protozoa Optimizer (APO)—a state-of-the-art bio-inspired metaheuristic proposed by Wang et al. [18] in 2024—to adaptively optimize DBSCAN’s Eps and MinPts parameters. This eliminates subjective empirical biases and enhances the framework’s generalization across complex scenarios. Second, we construct a multi-objective optimization function that integrates two critical metrics: an Euler solution residual penalty term (quantifying the reliability of inversion results) and a silhouette coefficient penalty term (evaluating clustering cohesion and separability). This design ensures that the optimized clustering results not only have high density but also align with the physical validity of Euler deconvolution—an integration rarely achieved in previous clustering-based methods. Third, by leveraging the APO’s balanced exploration (via autotrophy and hibernation behaviors) and exploitation (via heterotrophy and reproduction mechanisms), the framework achieves efficient parameter search and strong noise resistance, overcoming the adaptability limitations of DBSCAN with fixed manual parameters. Finally, the centroid of each optimized DBSCAN cluster directly corresponds to the spatial position of magnetic targets, realizing high-precision localization without additional post-processing.

## 2. Effective Statistical Screening and Cluster-Based Screening Algorithm Using APO-DBSCAN

### 2.1. Effective Statistical Screening

**Figure 1:**
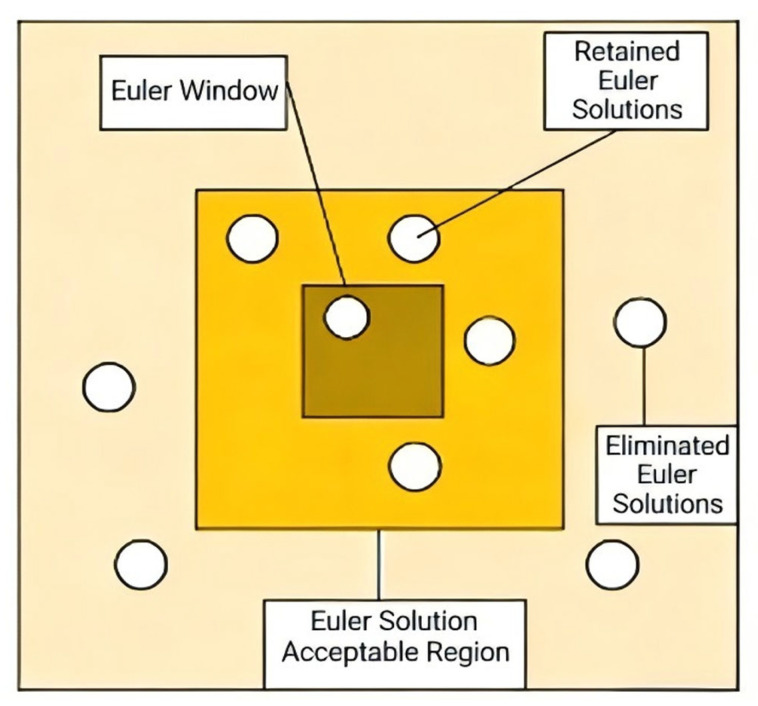
Schematic diagram of the effective region for Euler inversion results.

Anomaly Distance Criteria: Determine an appropriate acceptance zone for inversion results based on the horizontal relationship between the current Euler window and its inversion outcomes. This eliminates Euler solutions with significant deviations, reducing solution divergence to ensure positioning reliability. As shown in Figure 1, the dark-yellow area represents the Euler window region, the medium-yellow area denotes the acceptable zone for Euler solutions, and the “○” indicates potential Euler solution locations. When the Euler window is located near the target body, its inversion results exhibit smaller deviations and should fall within the Euler solution acceptable region horizontally. In such cases, the inversion results possess higher credibility and should be retained. Conversely, when the Euler window is positioned far from the magnetic target, its inversion results show larger deviations and will lie outside the Euler solution acceptable region horizontally. Under these circumstances, the inversion results have lower credibility and should be discarded.

Results from multiple Euler windows surrounding a magnetic target exhibit significant clustering characteristics. Such densely distributed Euler solutions are considered more reliable. Leveraging this characteristic, the criterion employs preset screening radii and Euler solution density thresholds to filter Euler solutions, discarding isolated spurious solutions. This approach reduces the divergence of Euler solutions, thereby enhancing the accuracy of magnetic target localization.

### 2.2. Classic DBSCAN Clustering Algorithm

The classic DBSCAN density-based clustering algorithm is an unsupervised learning method that defines cluster structures based on data point density. Its core logic is that if the density of data points within a region exceeds a preset threshold, these points are grouped into the same cluster. This algorithm utilizes neighborhood parameters to characterize the density distribution of data samples, representing a typical density-based clustering approach [19]. Unlike traditional distance-based clustering algorithms, DBSCAN performs clustering based on the density information surrounding each sample point. The resulting clusters are sets of data samples that satisfy maximum density connectivity, enabling the algorithm to recognize clusters of arbitrary shapes. During clustering, the algorithm first randomly selects a core point from the sample set as the starting point. It then completes clustering by combining preset parameters with neighborhood division rules [20]. DBSCAN characterizes the density state of sample distributions through two global parameters: the neighborhood radius, Eps, and the minimum number of points, MinPts. For any point in the sample space, the data point density is estimated by counting the number of other sample points contained within a neighborhood centered at that point with radius Eps.

**Definition** **1.** *The Eps neighborhood of any point p ∈ X in the object set X is defined as N_Eps_(p):*

(1) $$N_{Eps}(p)=\left\{q\in X|dist\left(p,q\right)<Eps\right\}$$

**Definition** **2** (core point)**.** 
$|N_{Eps}(p)|⩾MinPts$

**Definition** **3** (boundary point)**.** 
$1<\left|N_{\mathrm{Eps}}(p)\right|<\mathrm{MinPts}$

**Definition** **4** (noise point)**.** 
$\left|N_{Eps}(p)\right|=1$

**Definition 5** (Direct density reachability holds)**.**  *If point p is a core point and point q belongs to p’s Eps neighborhood N_Eps_(p), then the path from p to q satisfies direct density reachability.*

**Definition 6** (Density-reachable)**.**  *If for a sequence of points p_1_, p_2_, …, p_n_ exists in the object set, such that p_1_ = p, p_n_ = q, and p_i_ and p_i+1_ are density-reachable for each i in {1, 2, …, n − 1}, then the path from p to q is density-reachable.*

**Definition 7** (Density-connected)**.**  *If point p is density-reachable to another point o in the objects, and simultaneously point q is density-reachable to o, then p is density-connected to q.*

**Definition 8** (Cluster)**.**  *For any non-empty subset C of an object set X, C is a cluster if it satisfies the following:*

1. *For any p ∈ C and any q ∈ X, if q is density-reachable from p, then q ∈ C.*
2. *Any two points p, q in C satisfy either direct density reachability or density connectivity.*

**Figure 2:**
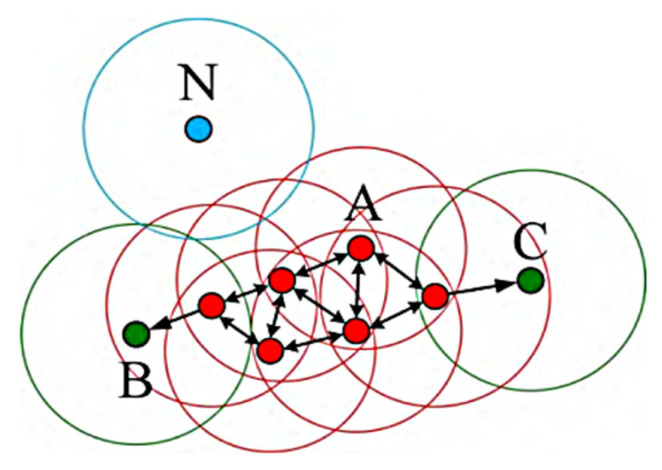
Schematic diagram of DBSCAN’s basic principles.

The basic DBSCAN process is as follows: ① Input global parameters Eps and MinPts. ② In the object set X, select any unclassified point *p* ∈ X and compute its Eps neighborhood *N*_Eps_(*p*) using Equation (1). Based on Definitions 3–5, if *p* is a core point, create a new cluster and iterate the algorithm starting from *p* to find all density-reachable or density-connected neighbors in the object set, assigning these points to the cluster. Otherwise, treat *p* as a noise point. ③ Repeat step ② until all non-noise points are classified. The principle of DBSCAN is illustrated in Figure 2, where the red point A represents a core point, the green points B and C represent density-reachable points, and the blue point *N* represents a noise point. The cloud data of red and green points form a point cloud cluster.

### 2.3. Artificial Protozoa Optimizer (APO) Optimization Algorithm

APO is an emerging bio-inspired heuristic algorithm proposed in April 2024 in the SCI Q1 journal *Knowledge-Based Systems* [18]. It primarily addresses complex engineering optimization problems. Inspired by natural protozoa, this algorithm demonstrates robust adaptability and search capabilities through unique foraging and survival strategies. In APO, whether protozoa engage in feeding (autotrophy and heterotrophy), hibernation, or reproduction depends on a proportional fraction (*f_p_*) [21]. Autotrophy and hibernation prioritize extensive coverage search, enhancing the algorithm’s exploration capability, while heterotrophy and reproduction enable rapid identification of optimal regions. The primary mathematical model of APO is

(2) $$f_{p}=f_{p,\max}\times rand$$

(3) $$p_{ah}=\frac{1}{2}\left(1+\cos\left(\frac{iter}{iter_{\max}}\times \pi \right)\right)$$

(4) $$p_{dr}=\frac{1}{2}\left(1+\cos\left(\left(1-\frac{i}{p_{s}}\right)\pi \right)\right)$$

In the equation, *iter* denotes the iteration count; *p_ah_* represents the probability of autotrophy and heterotrophy; and *p_dr_* denotes the probability of hibernation or reproduction.

### 2.4. DBSCAN Clustering Algorithm with APO-Optimized Parameters

DBSCAN identifies high-density regions separated by low-density areas based on clustering density, thereby achieving clustering. Compared to other distance-based clustering algorithms, DBSCAN can recognize clusters of arbitrary shapes in spatial databases and connect adjacent regions through density, making it suitable for processing spatial data. The discrete Euler solution computed via Euler deconvolution is susceptible to interference from non-target magnetic bodies and noise. This makes it challenging to pre-determine the neighborhood radius Eps and minimum point count MinPts during DBSCAN clustering of Euler solutions [22]. This paper pioneers the application of APO to optimize the DBSCAN parameter combination [Eps, MinPts].

In the context of unsupervised learning, the validity and reliability of clustering results are difficult to ascertain. Therefore, clustering metrics are proposed to evaluate their stability and accuracy. The objective function for APO optimization is introduced below.

In DBSCAN clustering optimization using Eulerian deconvolution, the objective function must simultaneously incorporate the Euler solution residual penalty term (reflecting the reliability of Euler solutions) and clustering quality constraints (compactness and separability). The objective function constructed in this paper adopts a minimization optimization approach, integrating three modules: the Euler solution residual penalty term and the contour coefficient penalty. The mathematical expression is as follows,

(5) $$\mathrm{fitness}=w_{n}\cdot P_{n}+w_{\mathrm{sil}}\cdot P_{\mathrm{sil}}$$

where $w_{n}$ and $w_{\mathrm{sil}}$ are weighting coefficients satisfying $w_{n}$ + $w_{\mathrm{sil}}$ = 1. These are set based on geological objectives and clustering requirements. (In this paper, parameters were experimentally validated and set to $w_{n}$ = 0.3 and $w_{\mathrm{sil}}$ = 0.7.)

$P_{n}$ is the Euler solution residual penalty term, penalizing clusters with low fitting accuracy in the Euler deconvolution process (smaller residuals indicate higher solution reliability); $P_{\mathrm{sil}}$ is the contour coefficient penalty term, measuring the comprehensive quality of cluster separation and cohesion;

Euler Solution Residual Penalty Term $P_{n}$:

(6) $$P_{n}=\frac{1}{N}\sum_{i=1}^{N}R_{i}$$

*R_i_* denotes the fitting error of the equation for the i-th Euler solution, and *N* represents the total number of filtered Euler solutions.

Contour coefficient penalty term $P_{\mathrm{sil}}$:

(7) $$P_{\mathrm{sil}}=1-\mathrm{Sil}$$

(8) $$\mathrm{Sil}=\frac{1}{N}\sum_{i=1}^{N}\frac{b(i)-a(i)}{\max(a(i),b(i))}$$

The silhouette coefficient is a classic metric for evaluating clustering quality, ranging from [−1, 1]. A value closer to 1 indicates that the clusters exhibit “high cohesion and good separation.” a(i) represents the average distance from the discrete Euler solution i to other solutions within the same cluster (cohesion: lower values indicate greater compactness). b(i) denotes the average distance from sample i to all solutions in the nearest dissimilar cluster (a cluster different from the one that solution i belongs to) (separation: higher values indicate greater separation). N is the total number of valid samples (excluding outliers).

**Figure 3:**
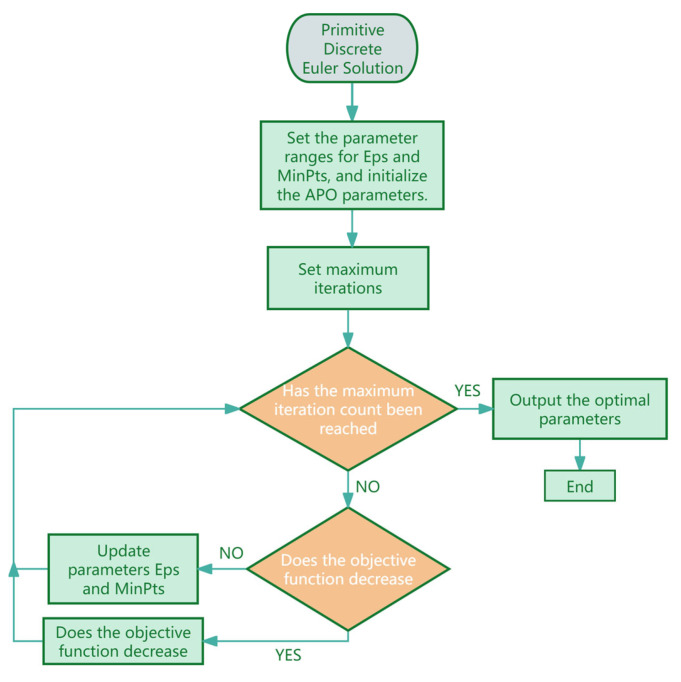
Workflow for APO-optimized DBSCAN parameter tuning.

As shown in Figure 3, the specific process for optimizing the DBSCAN algorithm using APO is presented.

## 3. Simulation Experiments

### Magnetic Sphere Models and Observation Grid Parameters

**Figure 4:**
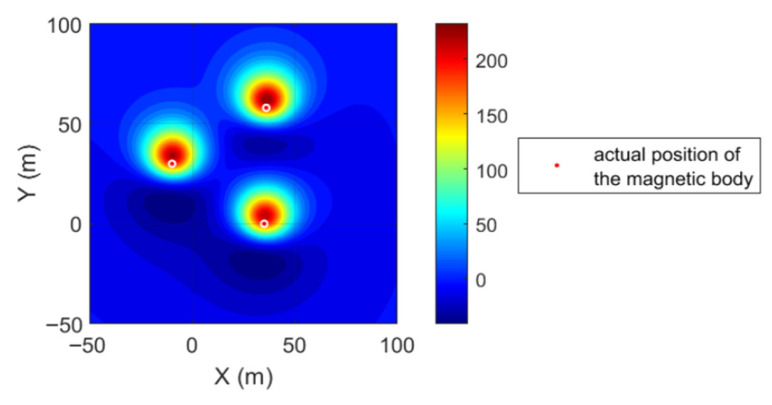
Magnetic anomaly contour map.

The experiment constructs three uniformly magnetized magnetic spheres as target bodies. All spheres have a radius of 3 m, with center coordinates set at (−10, 30, 20), (35, 0, 20), and (36, 58, 20). All spheres have a magnetic field strength amplitude of 102 A/m, with a magnetization dip angle of 45° and a declination angle of 5°. They were buried at a depth of 20 m (z-axis coordinate), spatially distributed across distinct X-Y plane regions to facilitate differentiation of independent anomaly characteristics. A regular two-dimensional grid was employed as the magnetic anomaly observation surface, with grid dimensions set to X-axis [−50, 100] m, Y-axis [−50, 100] m, with grid spacing dx = dy = 1 m, ultimately generating a 151 × 151 pixel observation grid (151 points in the X direction, 151 points in the Y direction). The magnetic anomaly contour map generated by the three spheres is shown in Figure 4.

**Figure 5:**
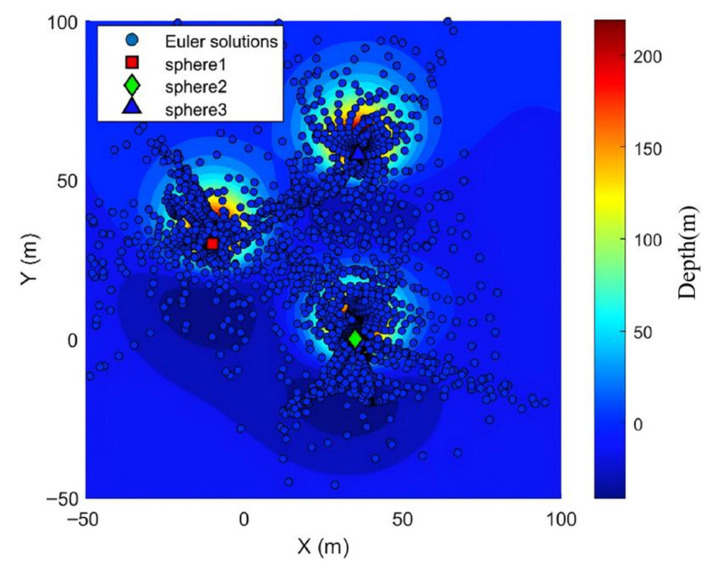
Planar distribution map of the original Euler solution.

**Figure 6:**
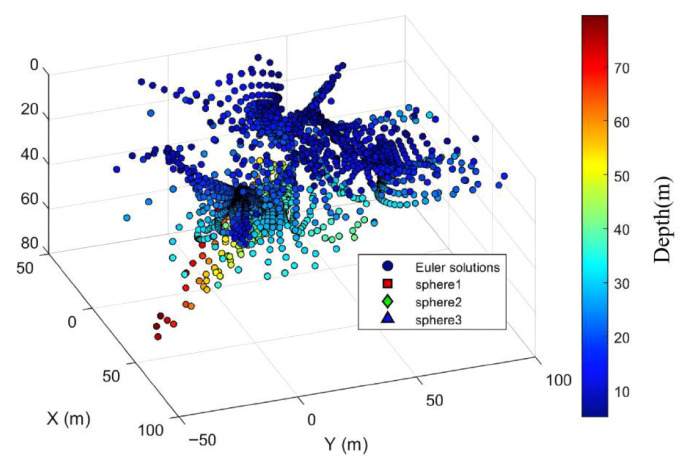
Three-dimensional distribution map of the original Euler solution.

The sliding Euler deconvolution method was directly applied to solve the magnetic anomaly map. The Euler window size was set to 11 m × 11 m, with a step size of twice the grid spacing. When solving magnetic target parameters using the Euler method, the horizontal and vertical derivatives of the magnetic field must be computed. Here, the finite difference method and the two-dimensional fast Fourier transform method are employed respectively. With the fixed structure index set to 3, the horizontal and vertical derivatives are substituted into the Euler field equations and solved using the least squares method. The planar distribution map and three-dimensional distribution of the original Euler solution are shown in Figure 5 and Figure 6.

Euler solutions exhibit spatial concentration and structural indices near magnetic targets, while showing dispersion in other regions. Quality screening of Euler solutions employs the most commonly used main anomaly distance criterion and Euler solution clustering criterion.

**Figure 7:**
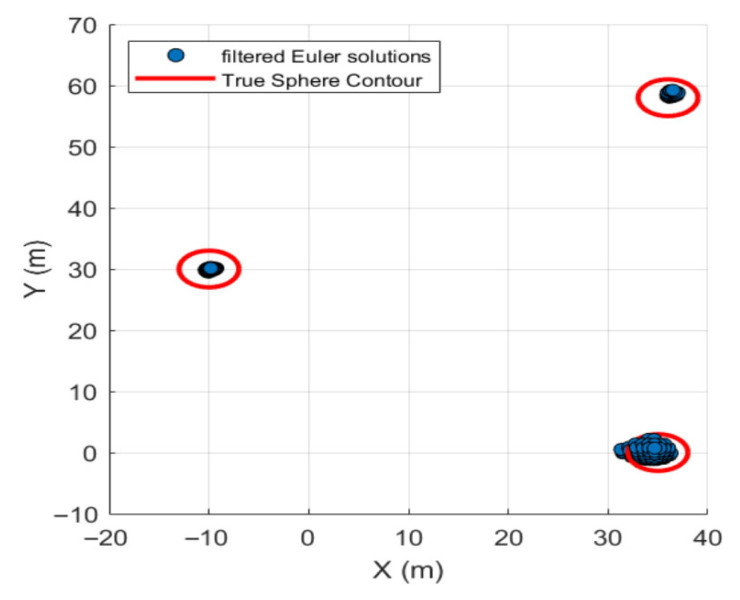
Planar view of discrete Euler solution after quality screening.

**Figure 8:**
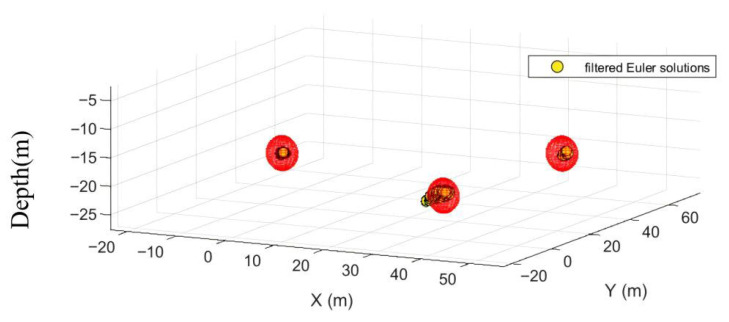
Three-dimensional view of the discrete Euler solution after mass screening.

Based on these two criteria, the inverted results with high clustering density are deemed reliable and should be retained; otherwise, they should be discarded. The final retained results correspond to magnetic targets within the observation area. The main anomaly distance threshold set based on these two criteria is 7 times the grid spacing (typically ranging from 5 to 10 times the grid point distance). The density radius for the Euler solution clustering divergence is 7 times the grid point distance, with a density threshold of 9. The planar and three-dimensional views of the quality-screened Euler solutions are shown in Figure 7 and Figure 8.

After screening based on the two major criteria, 3454 Euler solutions were eliminated. Among the retained solutions, 139 were valid (Euler solutions located inside the sphere) and 7 were invalid (Euler solutions located outside the sphere). However, Figure 7 and Figure 8 indicate that while these criteria effectively controlled solution divergence, they may have also reduced the number of valid solutions, suggesting potential over-screening.

Below, we employ the APO-optimized DBSCAN clustering algorithm proposed in this paper for Euler solution quality screening. The core parameters of the APO optimization algorithm are set as follows: population size is set to 25, balancing search accuracy and computational efficiency (excessively large populations increase computational time, while excessively small populations risk local optima); the maximum iteration count is set to 40. Testing indicates that the fitness function value stabilizes when iterations ≥30, and 40 iterations ensure complete algorithm convergence. The behavioral mode switching probability controls the algorithm to execute foraging behavior (core search process) in 80% of iterations and hibernation/reproduction behavior (to avoid local influence of global optimal solutions and neighboring individuals on the search direction, ensuring diversity and directionality in the search).

Meanwhile, the search ranges for the parameters are set as follows: the search range of Eps (Neighborhood Radius, representing the spatial correlation scale of magnetic anomaly signals and characterizing the spatial neighborhood scope for clustering valid Euler deconvolution solutions) is defined as [0.1, 2.0]; the search range of MinPts (Minimum Number of Neighborhood Points, the minimum number of valid Euler solutions required within an Eps neighborhood to identify a statistically significant and stable magnetic body cluster) is defined as [10, 80].

**Figure 9:**
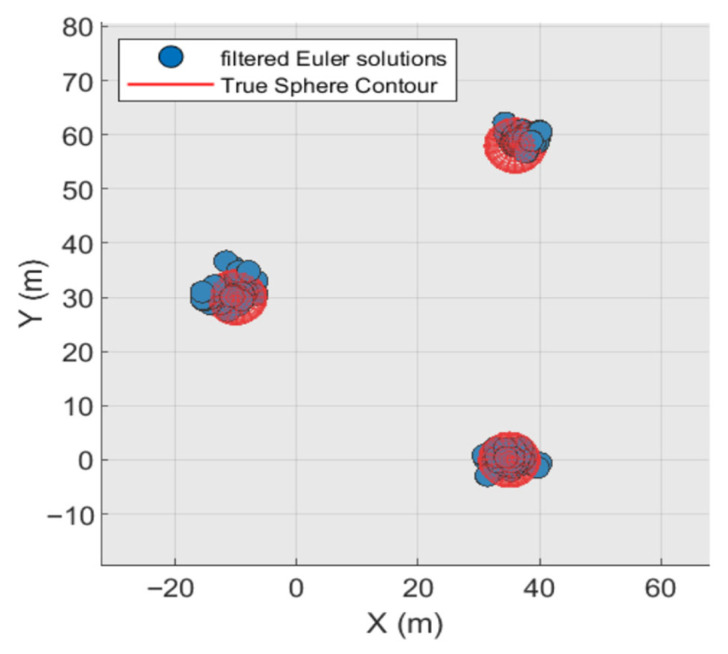
Planar view of discrete Euler solution after quality screening by optimization algorithm.

**Figure 10:**
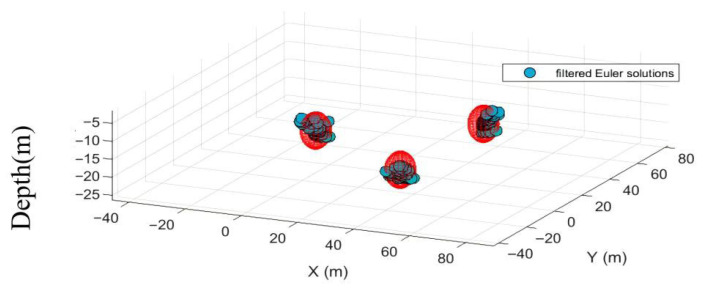
Three-dimensional view of the discrete Euler solution after quality screening by the optimization algorithm.

After forty iterations, the optimal EPS and MinPts parameters were obtained as 0.2353 and 42, respectively. The DBSCAN algorithm with these optimal parameters was applied for quality screening of Euler solutions, as shown in Figure 9 and Figure 10.

After filtering through an optimized algorithm, 3375 Euler solutions were discarded. Among the retained solutions, 212 were valid (Euler solutions located inside the sphere) and 5 were invalid (Euler solutions located outside the sphere). Compared to conventional statistical screening methods, this optimized approach intelligently filters out low-quality Euler solutions without manual intervention, preserving more valid solutions. The retention rate of valid solutions increased by 52.52%, while the retention rate of invalid solutions decreased by 28.57%. Simultaneously, the location of discrete solution clusters was determined using their centroids. Detailed data are presented in Table 1.

To validate the noise resistance of the new algorithm and simulate the random noise characteristics of magnetic anomaly observation data, zero-mean Gaussian white noise was superimposed on the magnetic anomaly signal of a pure magnetic sphere. The noise statistical parameters were defined as follows: the noise mean was set to 0 nT, and the standard deviation was set to 2 nT. Based on the size of the magnetic anomaly grid (consistent with the dimensions of the X and Y grid matrices), a noise matrix with matching dimensions was generated using a normal distribution random number generator. This noise matrix was then element-wise superimposed onto the pure magnetic anomaly signal to obtain the noisy magnetic anomaly data.

**Figure 11:**
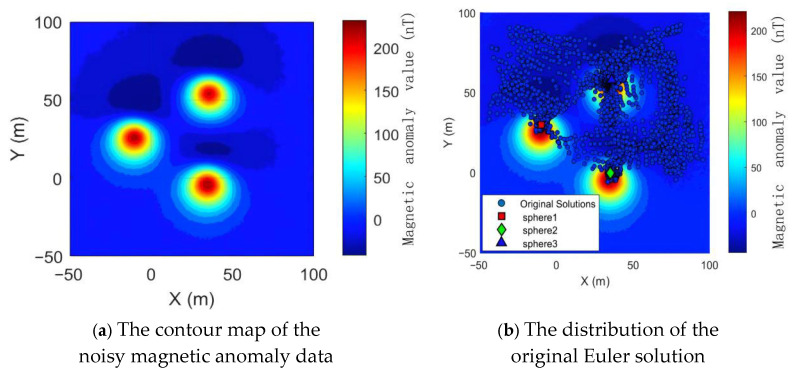
Contour map of magnetic anomaly data with noise and distribution of original Euler solutions.

The contour map of the noisy magnetic anomaly data are shown in Figure 11a, while the distribution of the original Euler solution after Euler deconvolution is presented in Figure 11b.

**Figure 12:**
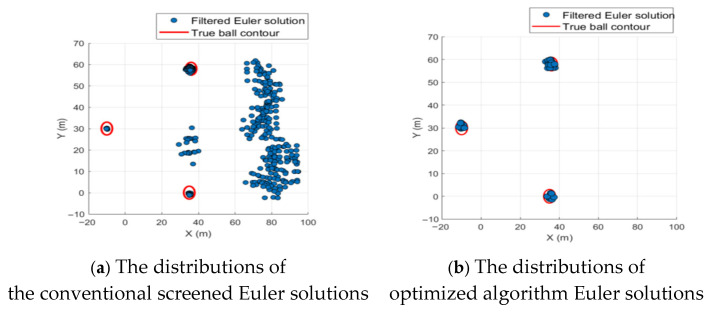
Distribution of Euler solutions under different screening methods.

Both the conventional screening criteria and the proposed optimized algorithm were used for quality screening of Euler solutions (all parameters identical to those described earlier). The distributions of the screened Euler solutions are shown in Figure 12a,b.

After effective statistical screening, 2431 Euler solutions were eliminated. Among the retained solutions, 131 were valid (Euler solutions located inside the sphere) and 294 were invalid (Euler solutions located outside the sphere). The figure clearly demonstrates that traditional statistical screening based on two major criteria is highly dependent on parameter selection when facing external noise interference. Inappropriate parameter selection can lead to the retention of numerous invalid solutions caused by noise, as shown in Figure 12a. In contrast, after screening with the optimized algorithm (Figure 12b), 2608 Euler solutions were eliminated. Valid solutions (Euler solutions located inside the sphere) were retained at 231 (a 76.33% increase in valid solution retention rate), while invalid solutions (Euler solutions located outside the sphere) were retained at 17 (a 94.21% decrease in invalid solution retention rate). This approach fundamentally eliminates most invalid solutions caused by noise and other disturbances, validating that the optimization algorithm proposed in this paper can still achieve excellent results even when data are affected by external noise and other factors.

## 4. Field Data Validation

This paper employs survey lines from the Panjin offshore magnetic survey project for algorithm evaluation. For the Hainan 8 block of the Liaodong Bay oilfield, located in the northern Liaodong Bay and the waters south of the Shuangtaizi River estuary, a main survey line was established with a spacing of 5 m, comprising 21 survey lines and a total length of 2.73 km. The entire project employed the MAG-885 cesium-optically pumped magnetometer (Shanghai Yangxiang Marine Technology Co., Ltd., Shanghai, China) for measurements. The navigation and positioning system comprised a Global Positioning System (GPS model: Trimble SPS356) and Hypack navigation software (HYPACK 2025).

**Figure 13:**
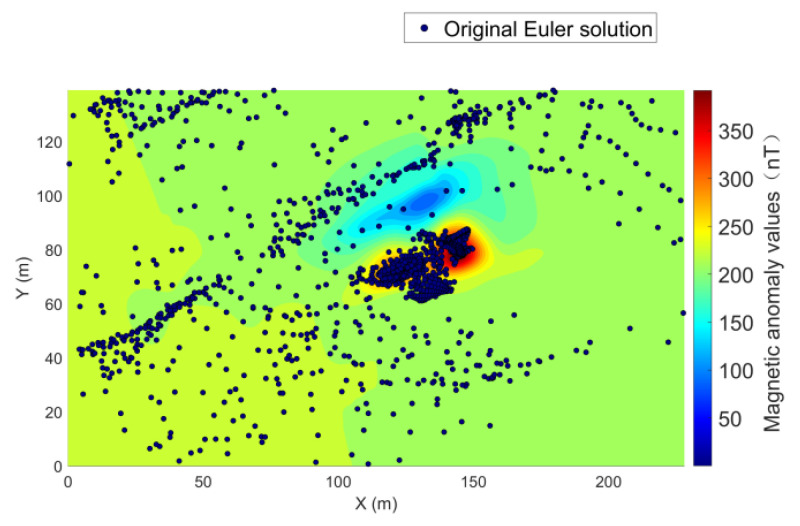
Distribution of original Euler solutions.

First, severe jump points that did not meet specifications were removed from the survey line data, and normal fields were eliminated. After gridding the survey line data (grid interval: 0.8 m), Euler deconvolution parameters were set based on prior information as follows: structural index = 2 (submerged magnetic objects assumed as horizontal cylindrical steel piles), sliding window size = 9 times the grid interval, and window step = 1. Euler deconvolution was applied to the data, yielding the initial Euler solution distribution shown in Figure 13.

**Figure 14:**
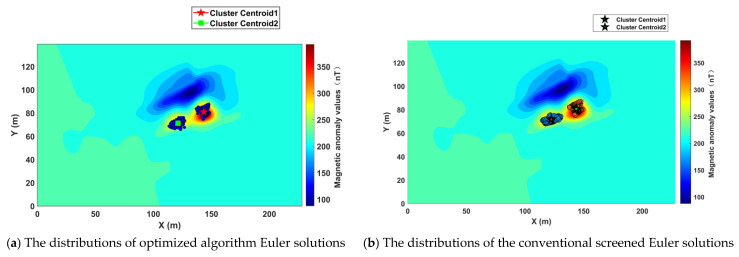
Euler solution after screening.

After applying optimization algorithms and effective statistical screening to the original Euler solutions, the filtered Euler solution distributions are as shown in Figure 14a,b.

Localization was achieved using the center of gravity of the discrete solution cluster. Detailed data are presented in Table 2.

As shown by the deviations in Table 2, the optimized algorithm proposed in this paper achieves higher accuracy in locating discrete Euler solution clusters after screening compared to the currently most widely used effective statistical screening method. The average deviation of the true center of gravity is reduced by 28.06%. (As can be seen from Table 2, the 3D centroid deviations of Cluster Centroid 1 and Cluster Centroid 2 are reduced by 1.12 m and 0.89 m, respectively, with the corresponding deviation reduction percentages being 27.60% and 28.52%, and the average reduction being 28.06%.) The reason for this may be that when confronted with a large number of invalid solutions generated by real-world measurement data containing various noise interferences, the optimization algorithm proposed in this paper can more effectively filter out invalid solutions while retaining more valid ones. This ultimately leads to more precise positioning of the center of gravity in the filtered solution clusters.

## 5. Conclusions

To address issues with traditional Euler solution quality control methods—such as excessive filtering of valid solutions, parameter settings reliant on subjective experience, and insufficient noise resistance—this study proposes a novel quality control approach for Euler deconvolution discrete solutions based on APO-DBSCAN clustering.

The algorithm was validated using simulation model data and magnetic survey data from underwater steel piles in marine areas. The key conclusions are as follows:

Simulation analysis and real-world data validation demonstrate that the proposed optimization algorithm exhibits superior robustness under external noise interference. Quantitative comparisons reveal the following: Compared to traditional effective statistical screening methods, this optimized algorithm increases the retention rate of valid solutions by 52.52% and 76.33% in noise-free and noisy datasets, respectively, while reducing the retention rate of invalid solutions by 28.57% and 94.21%. In practical application scenarios using real-world data, the average displacement of the target’s true center of gravity decreased by 28.06%, fully validating the algorithm’s significant advantages in enhancing solution validity and positioning accuracy.

The target optimization function, which integrates residual penalty terms for Euler solutions and contour coefficient penalty terms, addresses the spatial distribution characteristics of Euler solutions. This function balances the fitting reliability of Euler solutions while ensuring the cohesion and separation of clustering results, providing a quantitative basis for the precise screening of discrete solutions.

This study enhances the quality control system for discrete solutions in gravity-magnetic field inversion, offering theoretical and practical value applicable to multiple engineering fields such as underwater target detection. Future research may expand the algorithm’s applicability by optimizing the objective function for non-uniform magnetization and complex-shaped field sources, exploring the integration of the APO algorithm with other clustering models, and improving adaptability and interpretation accuracy in complex geological conditions.

## Figures and Tables

**Table 1:** Statistics on the actual positions of small balls and the distribution of center of gravity for discrete solutions after screening.

Number of Balls	A			B			C		
Actual location (/m)	x	y	z	x	y	z	x	y	z
	−10	30	20	35	0	20	36	58	20
Effective statistical filtering (/m)	−9.9	29.8	19.9	34.2	0	19.7	36.3	58.6	20.2
Deviation (/m)	0.1	0.2	0.1	0.8	0	0.3	0.3	0.6	0.2
Optimized Algorithm Screening (/m)	10.4	30.3	20.1	35.2	0.2	20.2	36.1	58.8	19.8
Deviation (/m)	0.4	0.3	0.1	0.2	0.2	0.2	0.1	0.2	0.2

**Table 2:** Statistics on the actual position of the magnetic target and the distribution of the center of gravity of the discrete solution after screening.

	Cluster Centroid 1			Cluster Centroid 2		
Actual location (/m)	x	y	z	x	y	z
	122.4	74.32	6.92	142.1	83.63	6.35
Effective statistical filtering (/m)	120.2	71.2	8.3	143.5	80.9	5.8
Deviation (/m)	2.2	3.12	1.38	1.4	2.73	0.55
Optimized Algorithm Screening (/m)	121.4	71.7	7.8	142.9	81.6	5.9
Deviation (/m)	1	2.62	0.88	0.8	2.03	0.45

## Data Availability

Data available upon reasonable request from the authors.
